# Short hairpin RNA of frizzled-2 suppresses the proliferation of hepatocellular carcinoma cells

**DOI:** 10.3892/ol.2014.2408

**Published:** 2014-08-04

**Authors:** MINORU TOMIZAWA, FUMINOBU SHINOZAKI, YASUFUMI MOTOYOSHI, TAKAO SUGIYAMA, SHIGENORI YAMAMOTO, MAKOTO SUEISHI

**Affiliations:** 1 Department of Gastroenterology, National Hospital Organization, Shimoshizu Hospital, Yotsukaido, Chiba 284-0003, Japan; 2 Department of Radiology, National Hospital Organization, Shimoshizu Hospital, Yotsukaido, Chiba 284-0003, Japan; 3 Department of Neurology, National Hospital Organization, Shimoshizu Hospital, Yotsukaido, Chiba 284-0003, Japan; 4 Department of Rheumatology, National Hospital Organization, Shimoshizu Hospital, Yotsukaido, Chiba 284-0003, Japan; 5 Department of Pediatrics, National Hospital Organization, Shimoshizu Hospital, Yotsukaido, Chiba 284-0003, Japan

**Keywords:** frizzled genes, quantitative PCR, MTS assays

## Abstract

In the present study, Frizzled-2 (Fz2), a receptor of the Wnt ligand, was investigated as a potential target of molecular therapy for hepatocellular carcinoma (HCC). Quantitative polymerase chain reaction (PCR) was performed to determine the expression levels of Fz2. A surgical specimen of HCC was immunostained with an Fz2 antibody. A 3-(4,5-dimethylthiazol-2-yl)-5-(3-carboxymethoxyphenyl)-2-(4-sulfophenyl)-2H-tetrazolium inner salt assay was performed on HCC cell lines, including HLF and Hep3B, 72 h after the transfection of the short hairpin (sh)RNA of Fz2 (shRNA-Fz2). RNA was isolated from the Hep3B and HLF cells 48 h after transfection and subjected to quantitative PCR. All cell lines had elevated levels of Fz2 compared with those in an adult liver. The highest and lowest expression levels of Fz2 were 246.9±15.7 in the HLF cells and 5.8±1.4 in the Hep3B cells, respectively. Fz2 was expressed in the tumorous HCC tissue, but not in the surrounding non-tumorous tissue. Cell proliferation was suppressed to 28.6±6.4% in the HLF cells and to 29.8±4.3% in the Hep3B cells at 100 ng shRNA-Fz2 per well. Levels of cyclin D1 expression decreased to 65.2±5.9% in the HLF cells and to 60.8±14.6% in the Hep3B cells at 2.5 μg per well. In conclusion, Fz2 was upregulated in the HCC cells. shRNA-Fz2 suppressed the proliferation of the Hep3B and HLF cells, decreasing Fz2 expression. As it was not expressed in the surrounding non-tumorous tissue, Fz2 may be an ideal molecular therapeutic target for HCC.

## Introduction

The Wnt pathway is involved in cell proliferation and differentiation (1). This pathway is divided into the canonical (β-catenin-dependent) and non-canonical (β-catenin-independent) pathways. The canonical pathway is activated when Wnt proteins bind to their receptor, frizzled (Fz), and co-receptor, low-density lipoprotein receptor-related protein 5 and 6, to form a complex (2,3). In the absence of Wnt signals, cytoplasmic β-catenin is maintained at low levels through destruction by the glycogen synthase kinase-3 β complex (4). β-catenin acts as a co-factor of T-cell factor/lymphoid enhancer factor and activates target genes. Constitutive activation of the Wnt pathway leads to abnormal cell growth and the development of cancer (5).

One of the non-canonical Wnt pathways is the planar cell polarity (PCP) pathway, whereby Fz activates a downstream pathway that includes small guanosine 5′-triphosphatases, ras-related C3 botulinum toxin substrate 1 and Ras homologue gene family member A (1). The PCP pathway is involved in cell polarity during morphogenesis (6). Another Wnt-Ca^2+^ pathway is one in which Wnt binds to Fz to activate heterotrimeric G proteins, leading to the activation of phospholipase C. The Wnt-Ca2^+^ pathway is involved in cancer (7–9). Wnt5a mediates the metastasis of malignant melanoma via the Wnt-Ca^2+^ pathway (10).

Structural and functional homology studies have identified 10 members of the Fz family of genes (11). Fz9 is involved in the canonical pathway (12) and is not expressed in the human fetal or adult liver (13). The short interfering RNA of Fz9 suppresses the cell proliferation and motility of hepatocellular carcinoma (HCC) and hepatoblastoma (HB) cell lines (13). Fz2 is involved in the non-canonical pathway, as it induces intracellular Ca^2+^ release (14). Reverse transcriptase polymerase chain reaction (PCR) results have shown that Fz2 is slightly expressed in the human fetal liver, but not in the adult liver (13). Fz2 is expressed in all HCC and HB cell lines. The role of Fz2 in liver carcinogenesis is, however, not completely understood.

The levels of Fz2 expression in HCC cell lines were therefore analyzed in the present study. The expression of Fz2 was suppressed with short hairpin (sh)RNA to clarify its role in the proliferation of HCC cell lines.

## Materials and methods

### Cell culture

The HCC cell lines, HLE, HLF, PLC/PRL/5, Huh-7, Hep3B, Huh-6, and HepG2, were purchased from RIKEN Cell Bank (Tsukuba, Japan). The cells were cultured in Dulbecco’s modified Eagle’s medium (DMEM) (Sigma-Aldrich, St. Louis, MO, USA) supplemented with 10% fetal bovine serum (FBS; Life Technologies, Grand Island, NY, USA). The cell lines were cultured with 5% carbon dioxide at 37°C in a humidified chamber. The study was approved by the ethics committee of the National Hospital Organization, Shimoshizu Hospital (Yotsukaido, Japan).

### Quantitative PCR

The cells were spread in 6-well plates (Asahi Techno Glass, Co., Ltd., Tokyo, Japan) and cultured. When they reached 80% confluency, the cells were further cultured for 48 h following transfection. Total RNA (5 μg), isolated with Isogen (Nippon Gene, Tokyo, Japan), was used to generate cDNA with Super Script III and oligo(dT) primers, following the manufacturer’s instructions (Life Technologies). Human fetal and adult liver RNA was purchased from Clontech Laboratories, Inc., (Mountain View, CA, USA). Quantitative PCR was performed using the Fast SYBR Green Master Mix (Life Technologies) and analyzed with the MiniOpticon Detection System (Bio-Rad, Hercules, CA, USA). The primer pairs for quantitative PCR and the resultant product sizes were as follows: Fz2 (NM_001466) forward, 5′-TCCTCAAGGTGCCATCCTATCTC-3′ and reverse, 5′-TGGTGACAGTGAAGAAGGTGGAAG-3′ (183 bp); cyclin D1 (NM_053056) forward, 5′-AGAGGCGGAGGAGAACAAACAG-3′ and reverse, 5′-AGGCGGTAGTAGGACAGGAAGTTG-3′ (180 bp); and ribosomal protein L (RPL)19 (BC095445) forward, 5′-CGAATGCCAGAGAAGGTCAC-3′ and reverse, 5′-CCATGAGAATCCGCTTGTTT-3′ (157 bp). Quantitative PCR was performed for 40 cycles of 5 sec of denaturation and 5 sec of annealing/extension. RPL19 was used as an internal control.

### Immunostaining

Sections of 38-week-old female human fetal liver, 64-year-old male adult liver and 60-year-old female HCC tissue (BioChain, Hayward, CA, USA) were deparaffinized, autoclaved and incubated, first with hydrogen peroxide and then for 30 min with 2% normal goat serum in phosphate buffered saline (PBS; washing buffer). Following overnight incubation with a rabbit polyclonal anti-Fz2 antibody (1:5,000; Sigma-Aldrich), specimens were rinsed with PBS and subsequently incubated with horseradish peroxidase-labeled anti-rabbit antibody (1:500) for 2 h (GE Healthcare, Pittsburgh, PA, USA). Next, diaminobenzidine (Dako, Glostrup, Denmark) was applied to the tissue sections as a chromogen and the nuclei were stained with hematoxylin (Muto Pure Chemicals Co., Ltd., Tokyo, Japan) for 15 sec. The specimens were observed and images were captured under an AX80 microscope (Olympus, Tokyo, Japan).

### Cell proliferation analysis

The cells were trypsinized, harvested, spread onto 96-well flat-bottom plates (Asahi Techno Glass Co., Ltd.) at a density of 1,000 cells per well, and then incubated for 24 h in DMEM supplemented with 10% FBS. Subsequent to being cultured, the cells were transfected with the shRNA of Fz2 (shRNA-Fz2) for 72 h. Cell cultures were subjected to 3-(4,5-dimethylthiazol-2-yl)-5-(3-carboxymethoxyphenyl)-2-(4-sulfophenyl)-2H-tetrazolium inner salt (MTS) assays, according to the manufacturer’s instructions (Promega Corporation, Madison, WI, USA). MTS is bio-reduced by cells into a colored formazan product that reduces absorbance at 490 nm. Absorbance was analyzed at a wavelength of 490 nm with an iMark Microplate Absorbance Reader (Bio-Rad).

### shRNA transfection

The shRNA-Fz2 (OriGene Technologies, Rockville, MD, USA) was transfected into the cells using Lipofectamine LTX (Life Technologies), according to the manufacturer’s instructions. Briefly, the shRNA was incubated with PLUS reagent for 5 min, following which, LTX reagent was added. A 30-min incubation at room temperature ensued, and the complex was subsequently applied to the cell culture medium. A negative control for shRNA was purchased from OriGene Technologies.

### Statistical analysis

Cell proliferation and quantitative PCR data were analyzed by a one-factor analysis of variance. Statistical analysis was performed using JMP 5.0 software (SAS Institute Inc., Cary, NC, USA). P<0.05 was considered to indicate a statistically significant difference.

## Results

**Figure 1:**
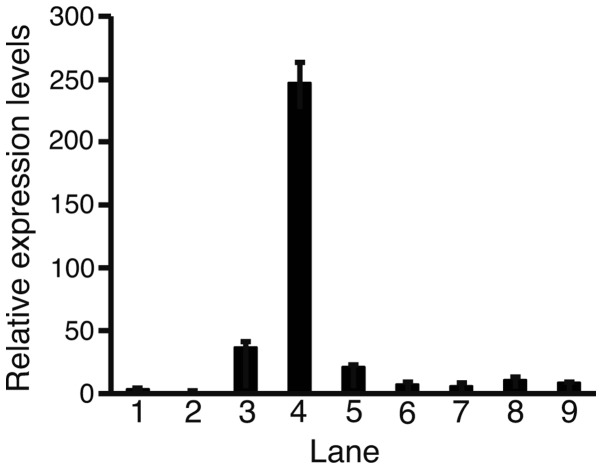
Quantitative polymerase chain reaction (PCR). Relative expression levels of frizzled (Fz)-2 were analyzed with quantitative PCR. Levels of Fz2 expression were normalized against those of the ribosomal protein L19. Each relative expression level of Fz2 was calculated as: Fz2 value/adult liver value. Lane 1, fetal liver; 2, adult liver; 3, HLE; 4, HLF; 5, PLC/PRL/5; 6, Huh-7; 7, Hep3B; 8, Huh-6; 9, HepG2; error bar, standard error.

Quantitative PCR was performed to determine the levels of Fz2 expression in the HCC cell lines compared with those in the fetal and adult liver cells (Fig. 1). All cell lines had elevated levels of Fz2 compared with the adult liver cells. The highest and lowest expression levels of Fz2 were 246.9±15.7 in the HLF cells and 5.8±1.4 in the Hep3B cells, respectively. These data suggest that Fz2 is upregulated in HCC cells.

**Figure 2:**
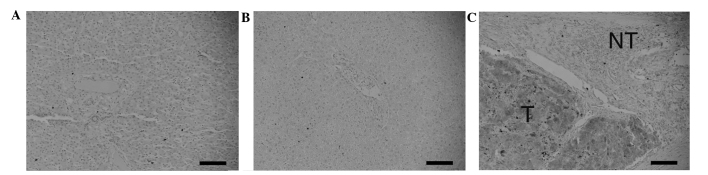
Immunostaining. Tissue specimens of (A) fetal liver, (B) adult liver and (C) hepatocellular carcinoma were immunostained with an antibody to frizzled-2. Original magnification, ×200; scale bar, 100 μm. T, tumorous tissue; NT, surrounding non-tumorous tissue.

Immunostaining was performed to clarify the expression levels of Fz2 at the protein level (Fig. 2). The fetal (Fig. 2A) and adult liver cells (Fig. 2B) did not express Fz2. By contrast, Fz2 was expressed in the tumorous HCC tissue (Fig. 2C). Fz2 was not expressed in the surrounding non-tumorous tissues. These data clearly indicate that Fz2 is upregulated at the RNA and protein levels.

**Figure 3:**
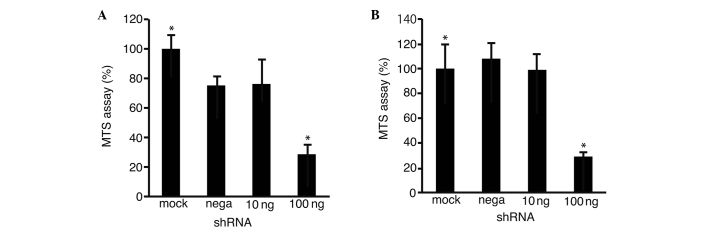
Transfection of short hairpin (sh)RNA to HLF and Hep3B cells. The shRNA of frizzled-2 was transfected into (A) HLF and (B) Hep3B cells, which were subjected to 3-(4,5-dimethylthiazol-2-yl)-5-(3-carboxymethoxyphenyl)-2-(4-sulfophenyl)-2H-tetrazolium inner salt (MTS) assays. Mock, mock transfection without any plasmid; nega, transfection of negative control of 10 ng shRNA; ^*^P<0.05 vs. mock transfection (n=3).

To clarify the possibility that Fz2 was involved in cell proliferation, an MTS assay was performed on the HLF (Fig. 3A) and Hep3B (Fig. 3B) cells transfected with the shRNA-Fz2. Cell proliferation was suppressed to 28.6±6.4% (P<0.05 vs. mock transfection) in the HLF cells and to 29.8±4.3% (P<0.05) in the Hep3B cells at 100 ng shRNA-Fz2 per well.

**Figure 4:**
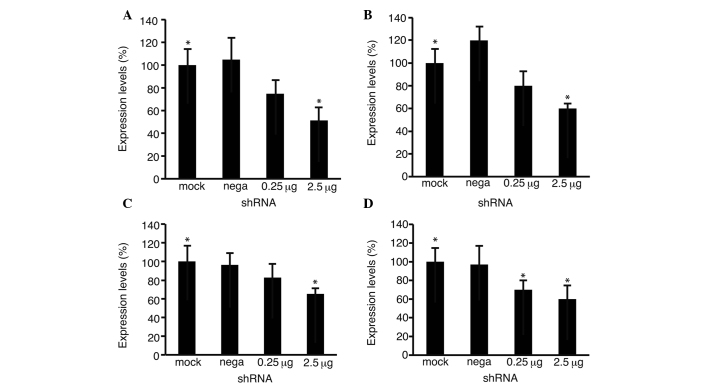
Quantitative polymerase chain reaction. Short hairpin (sh)RNA of frizzled-2 was transfected into (A and C) HLF and (B and D) Hep3B cells. RNA was isolated from the transfected cells and quantitative PCR was performed to analyze the expression levels of (A and B) frizzled-2 and (C and D) cyclin D1. Mock, mock transfection without any shRNA; nega, transfection of negative control of 2.5 μg shRNA; ^*^P<0.05 vs. mock transfection (n=3).

Quantitative PCR was performed to analyze the levels of Fz2 (Fig. 4A and B) and cyclin D1 (Fig. 4C and D) expression. The expression levels of Fz2 decreased to 51.3±10.7% (P<0.05 vs. mock transfection) in the HLF cells and 58.9±4.4% (P<0.05) in the Hep3B cells at 2.5 μg per well, confirming the effectiveness of shRNA-Fz2. The expression levels of cyclin D1 decreased to 65.2±5.9% (P<0.05) in the HLF cells and 60.8±14.6% (P<0.05) in the Hep3B cells at 2.5 μg per well.

## Discussion

Certain types of Fzs are upregulated in HCC. Fz3, 6, and 7 are upregulated in the tumorous tissues of HCC (15) and Fz2 is expressed in Hep3B (16). In the present study, quantitative PCR demonstrated that Fz2 was expressed to a greater degree in the HCC and HB cell lines than in the adult liver. The finding that Fz2 was more frequently upregulated in the tumorous tissues than in the non-tumorous tissues was expected. Immunostaining confirmed this hypothesis, showing that Fz2 was upregulated in the tumorous tissue more so than in the non-tumorous tissue from a surgical specimen of HCC. Systematic analysis of surgical specimens would be necessary to confirm this hypothesis. Immunostaining and western blot analysis have shown that Fz2 is upregulated in pancreatic cancer (17,18). The possible involvement of Fz2 in pancreatic cancer and HCC was expected.

Fz2 may be involved in cell proliferation. shRNA-Fz2 has been shown to suppress the proliferation of MIA-PaCa2, a pancreatic cancer cell line (17). In the present study, shRNA-Fz2 suppressed the proliferation of the HCC cell lines. shRNA-Fz2 suppressed the proliferation of the HLF cells, which had a 246.9±15.7-times higher expression level of Fz2 compared with the adult liver cells. The level of cyclin D1 expression is downregulated in MIA-PaCa2 cells (17). The present data clearly show that cyclin D1 was downregulated in the HLF and Hep3B cells. The downregulation of cyclin D1 may be one cause of the suppression of cell proliferation. The present data indicate that Fz2 may be a suitable molecular therapeutic target for HCC. One of the merits of Fz2 is that it was not expressed in the tumorous tissue of pancreatic cancer or HCC (17). The suppression of Fz2 expression was expected to have no effect on the surrounding non-tumorous tissues.

The next step would be to show apoptosis in the suppression of HCC cell proliferation with shRNA-Fz2. Another option would be to combine the shRNA-Fz2 treatment with other anticancer reagents (19,20).
